# A Virtual Out-of-Body Experience Reduces Fear of Death

**DOI:** 10.1371/journal.pone.0169343

**Published:** 2017-01-09

**Authors:** Pierre Bourdin, Itxaso Barberia, Ramon Oliva, Mel Slater

**Affiliations:** 1 Event Lab, Department of Clinical Psychology and Psychobiology, University of Barcelona, Barcelona, Spain; 2 Department of Cognition, Development and Educational Psychology, University of Barcelona, Barcelona, Spain; 3 Institució Catalana de Recerca i Estudis Avançats (ICREA), Barcelona, Spain; 4 Department of Computer Science, University College London, London, United Kingdom; University of Exeter, UNITED KINGDOM

## Abstract

Immersive virtual reality can be used to visually substitute a person’s real body by a life-sized virtual body (VB) that is seen from first person perspective. Using real-time motion capture the VB can be programmed to move synchronously with the real body (visuomotor synchrony), and also virtual objects seen to strike the VB can be felt through corresponding vibrotactile stimulation on the actual body (visuotactile synchrony). This setup typically gives rise to a strong perceptual illusion of ownership over the VB. When the viewpoint is lifted up and out of the VB so that it is seen below this may result in an out-of-body experience (OBE). In a two-factor between-groups experiment with 16 female participants per group we tested how fear of death might be influenced by two different methods for producing an OBE. In an initial embodiment phase where both groups experienced the same multisensory stimuli there was a strong feeling of body ownership. Then the viewpoint was lifted up and behind the VB. In the experimental group once the viewpoint was out of the VB there was no further connection with it (no visuomotor or visuotactile synchrony). In a control condition, although the viewpoint was in the identical place as in the experimental group, visuomotor and visuotactile synchrony continued. While both groups reported high scores on a question about their OBE illusion, the experimental group had a greater feeling of disownership towards the VB below compared to the control group, in line with previous findings. Fear of death in the experimental group was found to be lower than in the control group. This is in line with previous reports that naturally occurring OBEs are often associated with enhanced belief in life after death.

## Introduction

A Near-Death Experience (NDE) is an altered state of consciousness that can occur during clinical death—typically following cardiac arrest [1]. Characteristics of NDEs can vary widely, but generally include the perception of moving through a tunnel, bright lights, meeting spiritual beings, a panoramic life review, euphoria, and an out-of-body experience (OBE) [2–5]. Parnia, Waller, et al. [1] reported that 11% of cardiac arrest survivors experienced a NDE (n = 63), Van Lommel, van Wees, et al. [6] reported 18% (n = 344) and Klemenc-Ketis [7] reported 19% (n = 37). Generally patients exhibit a change in life outlook following such an event becoming more concerned about others, becoming more generous and charitable, and so on [7, 8]. Van Lommel, van Wees, et al. [6] found that this may take several years to consolidate, and indeed lasts several years after the event.

In this paper we concentrate only on the OBE aspect of a NDE. Blanke, Landis, et al. [9] defined an OBE “as the experience in which a person seems to be awake and to see his body and the world from a location outside the physical body”. In fact, OBEs are considered to be one of the key phenomenological characteristics of NDEs [2], and it has been found that people experiencing NDEs show reduced fear of death and enhanced belief in life after death [6]. The goal of the experiment reported in this paper is to examine whether an experience of the centre of perception of people being located outside their body might influence their fear of death. Metzinger [10] argued that the experience of OBEs may be the origin of the idea of the soul, i.e., survival after death. Our idea was that if we could put people in a situation illustrating the possibility of their consciousness being outside of their body, then this would provide implicit evidence (but not necessarily explicit belief) that survival beyond the body is possible, and hence produce a reduction in fear of death.

We used immersive virtual reality in order to accomplish this, and in particular the method of virtual embodiment. A person wearing a wide field-of-view head tracked stereo head-mounted display (HMD) can look in any direction within the virtual environment. In particular they can look towards their real body, and if the system has been so programmed they would see a life-sized virtual body visually substituting their own. Similarly they can see a reflection of the body in a virtual mirror and also shadows. With real-time motion capture the virtual body can be programmed to move synchronously with the person’s real body movements. Moreover, if something is seen to touch the virtual body, the setup can include vibrotactile stimulation on the person’s real body synchronous with the seen touch. Such multisensory stimulation typically leads to the perceptual illusion in people that the virtual body is their own, even though they know that this is not the case. This is referred to as a ‘full body ownership illusion’ (or just ‘body ownership illusion’) [11].

The idea is derived from the rubber hand illusion (RHI) first presented by Botvinick and Cohen [12] where a rubber hand is placed on a table in front of a person and seen to be tapped and stroked synchronously with corresponding stimulation on their out-of-sight real hand. We refer to this as synchronous visuotactile stimulation. This results in an illusion of ownership over the rubber hand, with physiological and brain activation anxiety responses when the rubber hand is attacked [13, 14]. When the stimulation is asynchronous the illusion does not occur. Petkova and Ehrsson [15] showed that tapping and stroking the abdomen of a manikin body seen through a HMD from first person perspective (1PP) as substituting the real body, while synchronously tapping and stroking correspondingly the real body, results in a full body ownership illusion over the manikin body. Similarly as in the case of the RHI, if the manikin body is attacked there is a skin conductance response indicating arousal, which occurs to a much lesser extent than when the visuotactile stimulation is asynchronous.

Slater, Perez-Marcos, et al. [16] showed that the RHI can be reproduced in virtual reality, where instead of a rubber hand a virtual arm and hand is seen as protruding out of the participant’s real body, and it was later shown that there is a corresponding cortical activity in response to a threat [17]. A further study [18] showed that a full body ownership illusion can also be achieved in virtual reality, where participants saw from 1PP a virtual body spatially coincident with and substituting their own, and also in a mirror, with visuotactile stimulation, and synchronous head movements. Since then a number of studies and experiments have reproduced the body ownership illusion in virtual reality—for example, investigating factors that lead to or break the illusion [19–21], and the implications of virtual body types for changes in attitudes and behaviours [22–24]. For a general review see [25, 26].

**Fig 1:**
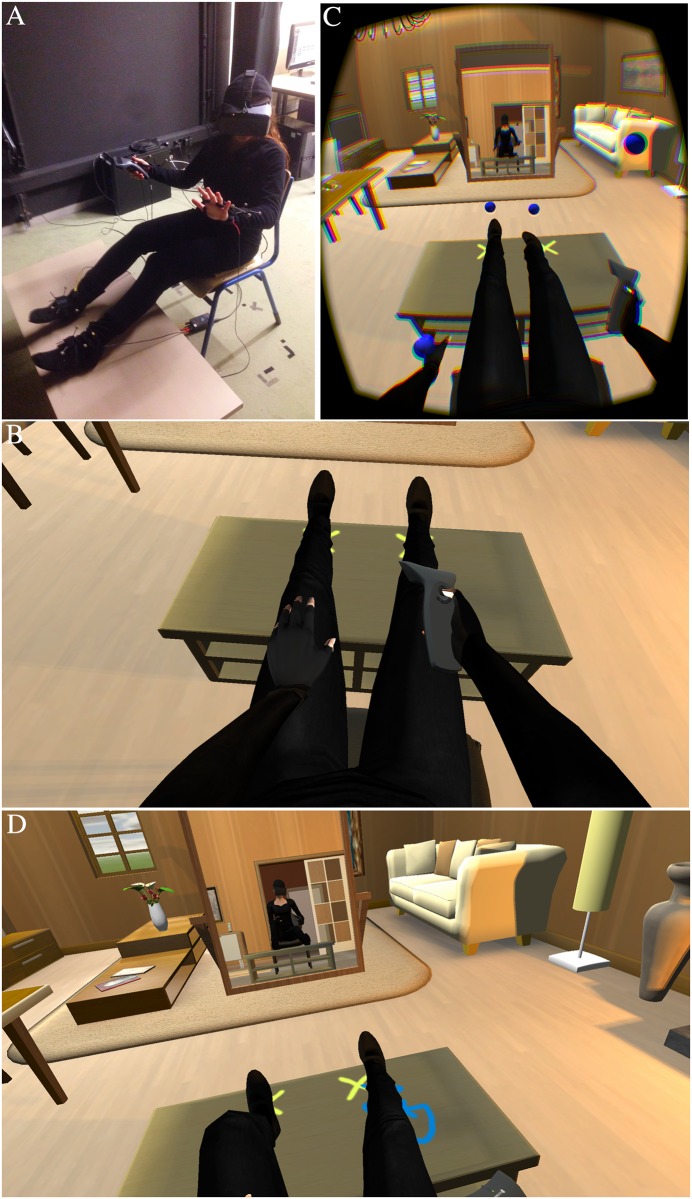
The overall setup and in-the-body phase. (A) A participant wearing the head-mounted display and body tracking suit with the tracking markers, and holding a Wand in the right hand. (B) Participants see their virtual body from first person perspective, including holding the Wand. (C) The virtual balls begin to strike the body with synchronous visuotactile stimulation, a reflection can be seen in the mirror in front. (D) The participant traces curves on the desk with the left or right foot, illustrating visuomotor synchrony.

The overall strategy we used was to first embody participants in a virtual body. In order to induce a strong body ownership illusion the virtual body was seen in 1PP and approximately spatially coincident with the real body and seen from the position of the eyes of the virtual body. The body was in a reclining position on a comfortable chair located in a virtual room. There was synchronous visuomotor correlation between real and virtual body movements, and visuotactile synchronous correlation—between virtual balls seen to be striking the body and vibrotactile stimulation on the corresponding body part. The method used was similar to [19] except that in this case the tactile stimulation was automatic rather than manual. The setup is shown in Fig 1. We refer to this as the ‘*in-the-body*’ phase. After a maximum of 11 minutes of this in-the-body phase, varying only because of the differing time participants needed to learn a mental ball-dropping task, participants experienced an *out-of-body* phase.

Ehrsson [27] induced the illusion of being out-of-body where participants saw their real body sitting in front of where their viewpoint was located. This was achieved by participants wearing a stereo HMD, to which video was streamed from a pair of cameras behind them. While they saw themselves from behind, the experimenter tapped their (out-of-sight) chest while synchronously striking under the camera with a stick. Hence from the viewpoint of participants it seemed as if the stick striking under the camera (i.e., under their viewpoint) were causing the feeling on their chest. The effect of this was to move their sense of perception behind where they saw their real body to be, resulting in an out-of-body illusion, where their real body was disowned, considered as an empty shell.

**Fig 2:**
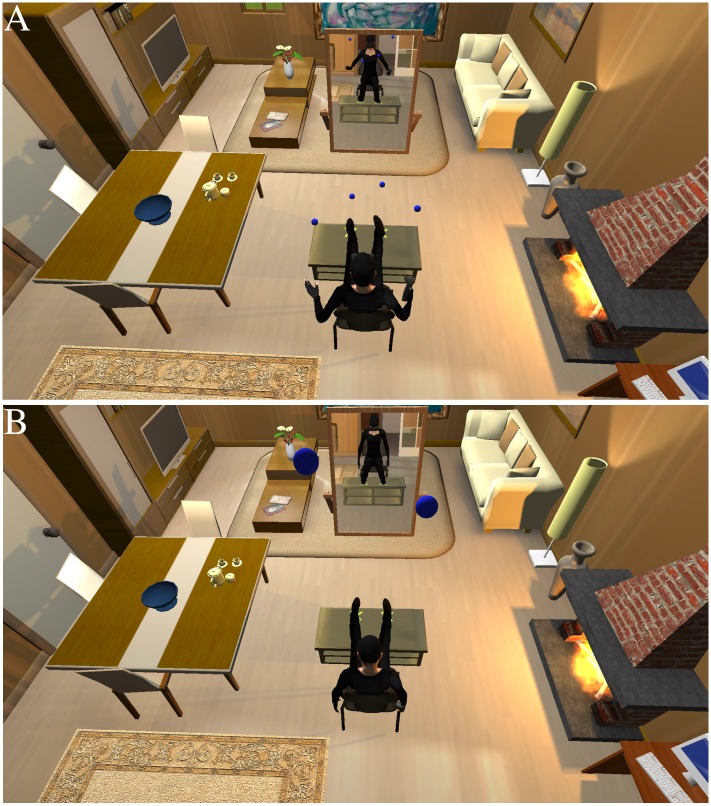
The out-of-body phase. (A) The DBE condition where the virtual balls continue to strike the body seen below and real body movement is mapped onto the virtual body. (B) The OBE condition where the balls strike where the body would be in relation to the elevated viewpoint, and real body movement does not affect the virtual body.

In the version that we used in this experiment, after the initial embodiment phase the viewpoint of participants moved up to the ceiling and behind the virtual body (Fig 2) and the virtual balls continued to strike the space around their visual centre of perception, i.e., where their body would have been, and correspondingly they felt the vibrotactile synchronous stimulation (Fig 2B). If they moved their real body, the virtual body below remained stationary. Hence visual and visuotactile sensations shifted to the location near the ceiling. We refer to this as the OBE condition. The goal in this condition was that participants would feel out of their body (the body in which they had previously been embodied with a high level of body ownership). Although in our method participants were shifted behind and upwards, compared to Ehrsson [27] where they were shifted only behind the real body, it has been shown that this makes no difference [28].

We used as a control condition (Fig 2A) another method based on [29]. This is almost the same as above, with participants seeing their body from behind, but seeing it tapped on the back while they feel this synchronously on their actual back. In this case perception is split between a visual point of view that is behind their real body, but also visual capture of the tapping seen on the back of the body in front leads to the feeling that the tactile sensation is located at the body in front. So while participants may feel out of their body they also have the sensation of being drawn towards the body in front, which manifests itself in a measurable drift in their estimated own body location towards the body in front. It was argued by Pomes and Slater [30] that the brain tries to resolve the contradiction between visual and visuotactile perception by unifying the two bodies (the body at the centre of visual perception and the remote body to which the visuotactile sensation is ascribed) by the illusion of drifting towards the body in front, or the virtual body drifting back towards the position of the participant.

Whereas in the method of Ehrsson [27] participants feel that they are out of their body with disownership of the body in front [31], the method of Lenggenhager, Tadi, et al. [29] results in a feeling of ownership over the body in front. We will call this method Drifting Body Experience (DBE).

Lenggenhager, Mouthon, et al. [32] carried out a study using both paradigms, with the participant’s viewpoint always raised above the surrogate body, and confirmed that OBE resulted in disownership of the surrogate body (the one they saw below), whereas DBE resulted in ownership. Moreover, when participants were asked to imagine dropping a ball and estimating the time before it hit the ground, their estimate tended to be a longer time in the OBE condition compared to the DBE. Our version of DBE, as for OBE, included a period when the viewpoint of the participant was lifted above and just behind the virtual body. However, in the DBE condition the balls continued to strike the virtual body seen below, and the participant felt corresponding vibrotactile stimulation synchronously. If the participant moved then the virtual body below would move synchronously. The overall experiment is shown in S1 Video.

Our experiment used a between groups design with n = 16 participants in each of the two groups (DBE and OBE). One group experienced body ownership while ‘in’ the virtual body as described above, and then experienced the OBE condition, and the other group the same in-the-body phase followed by the DBE condition. The second phase lasted for 1 m 23 s in both conditions, and involved seeing their virtual body from above as in Fig 2. Both groups consisted of women students recruited from the Faculty of Psychology at the UB campus with mean (± SD) age 20.3 ± 2.1. The groups were almost identical with respect to religious conviction and self esteem (see Section A in S1 File for comparisons between the two groups).

The study was approved by and carried out in accordance with the regulations of the Comisión de Bioética de la Universitat de Barcelona, and was therefore performed in accordance with the ethical standards laid down in the 1964 Declaration of Helsinki. Participants gave written informed consent on a form devised for this purpose that had been approved by the said Comisión de Bioética. They received course credits for participating in the study. The individual shown in Fig 1 and S1 Video of this manuscript has given written informed consent (as given in the PLOS consent form) to publish these. The information sheet, consent form, and all questionnaires were in Spanish and this paper only presents the English versions.

## Results

### Response Variables

The questions included in the questionnaire were inspired by questions from other studies—e.g., [18, 32]. Two questions investigating body ownership were asked after the initial in-the-body phase, by reading these out to participants and recording their score. Answers were on a Likert scale ranging from 1 to 7 with a value of 1 meaning they did not feel that at all, and a value of 7 meaning they felt it in its maximum intensity. Table 1 shows the questions, with response variables denoted *mybody* and *otherbody*.

**Table 1:** Body ownership and out-of-the body questions.

Variable Name	Questions on a 1–7 Likert Scale, 1 = ‘I did not feel that at all’, 7 = ‘I felt it to the maximum intensity’.
Immediately after the *in-the-body* phase: Please indicate to what extent you felt each of the sensations that I will indicate now.	
*mybody*	I felt as if the body I was seeing was my own body.
*otherbody*	I felt as if the body I was seeing belonged to someone else.
Immediately after *the out-of-body* phase: Please indicate to what extent you felt each of the sensations that I will indicate now. When you answer these questions, please refer to your experience when you were watching the room from above.	
*mybodyobe*	I felt as if the body I was seeing was my own body.
*otherbodyobe*	I felt as if the body I was seeing belonged to someone else.
*floatingobe*	I felt as if I was floating in air.
*elevatedobe*	I felt as if I was in an elevated position in the room.
*connectionobe*	I felt a connection with the body, as if I was looking down at myself.
*invisibleobe*	I felt as if I had an invisible body.
*obe*	I felt out of my body.

There were four questions asked immediately after the out-of-body phase, shown as *mybodyobe*, *otherbodyobe*, *connectionbodyobe*, and *obe*. The other questions were to test whether the condition was actually working (*floatingobe*, *elevatedobe*)—i.e., whether participants actually felt as if they were high up in the virtual room, and *invisibleobe* was for curiosity, whether participants would experience an invisible body given the findings reported in [33].

To evaluate the spatial self-location of participants, we adapted the mental ball-dropping task of Lenggenhager, Mouthon, et al. [32] asking participants to imagine how long it would take for an imaginary apple dropped from their hand to reach the floor. We used dropping of an apple rather than a ball to avoid confusion with the balls striking the virtual body. However, we still refer to this as a ‘mental ball-dropping task’. As in the original mental ball-dropping task, participants had to click a button of the Wand twice, first when releasing the imaginary apple and second when they estimated that the apple would reach the floor. At the start of the in-the-body phase participants were trained in this mental ball-dropping task, and then at the end of this phase they carried out this task 5 times each signaled by an auditory cue and separated by 3 s. The mean of these 5 drop times is denoted as *drop1*. This procedure was repeated at the end of the out-of-body phase, and the mean is denoted as *drop2*.

After the conclusion of the virtual reality exposure participants were asked to complete a subscale (‘Death of Self’) of the Collett-Lester Fear of Death scale designed to measure fear of death [34, 35], Spanish adaptation by Tomás-Sábado, Limonero, et al. [36]. The questions and corresponding response variables are shown in Table 2. The overall scores is the sum of the individual response items, denoted as FOD.

**Table 2:** The Collett-Lester Fear of Death Sub Scale.

Variable Name	Questions on a 1–5 Likert scale (1 = not at all, 5 = very much)
What level of worry or anxiety do you have for the following aspects related to your own death?	
solitude	The total isolation of death
lifeisbrief	The shortness of life
loseall	Missing out on so much after you die
dieyoung	Dying young
howitwillbe	How it will feel to be dead
candonothing	Never thinking or experiencing anything again
disintegration	The disintegration of your body after you die.

### Statistical Model

The formal (Bayesian) statistical model used is described in Section B in S1 File and uses the same approach as in [37]. We consider the response variables: body ownership (*mybody*, *otherbody*), out-of-body questions (*connectionobe*, *otherbodyobe*), mental ball drop times (*drop1*, *drop2*) and the fear of death questions of Table 2. For the out-of-body, mental ball drop, and fear of death questions the model uses the condition (DBE = 0, OBE = 1) as the independent variable. It should be noted that this is one overall model, where all stochastic equations are treated simultaneously rather than as a series of separate analyses—the Bayesian method returns the joint posterior distribution of all the model parameters. Note that all prior distributions on the model parameters were chosen to be non-informative, that is, with very large variance and heavily biased against our hypotheses. Analysis was carried out using the JAGS system [38], together with MATLAB using MATJAGS (http://psiexp.ss.uci.edu/research/programs_data/jags/), and some graphs were produced using Stata 14. The original data is provided in S1 Data.

### Body Ownership (in-the-body)

In order for our method to be valid we need to show that overall body ownership is high, the same for both groups, and that the responses to the *mybody* question are much greater than to the *otherbody* question.

**Fig 3:**
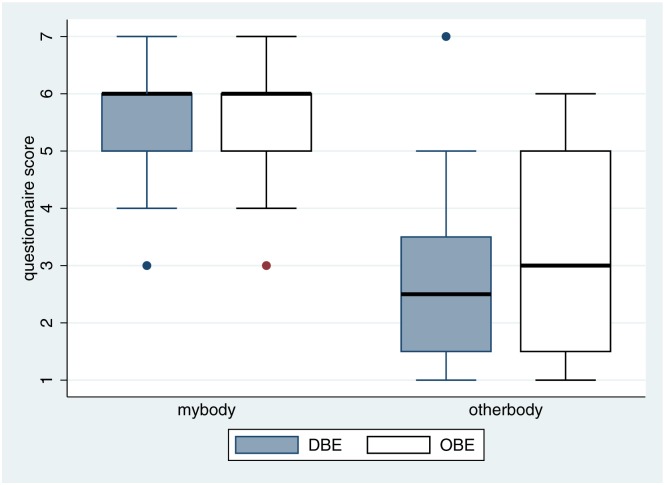
Box plots of the body ownership questionnaires by condition. The thick black horizontal lines are the medians, the boxes are the interquartile ranges and the whiskers extend from the minimum value (or the lower quartile—1.5*IQR) to the maximum value (or the upper quartile + 1.5*IQR). Values outside of this range are shown as single points.

Box plots of the responses to these questions are shown for each condition in Fig 3. It is clear that the overall response to *mybody* is high, with for each condition, 50% of participants giving a score of 6 or more, with at least 75% giving a score of 5 or more. The scores for *otherbody* are much lower. There are clearly no differences in the scores between the two conditions, as should be the case, since the in-the-body phase was the same for both groups. The results are similar to earlier ones—for example [39].

**Fig 4:**
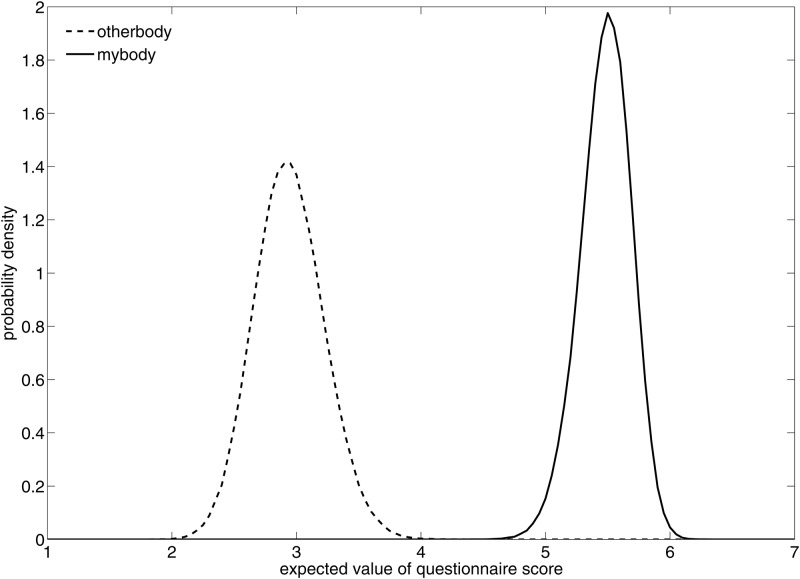
Posterior probability distributions of the expected values of *otherbody* and *mybody*.

The overall statistical model includes a logistic analysis of the ordinal questionnaire scores (*mybody*, *otherbody*). From this we can obtain the posterior distributions of the expected values (means) of *mybody* and *otherbody*. Fig 4 shows that the posterior distributions of the expected values of *mybody* and *otherbody* hardly intersect. The 95% credible intervals for *otherbody* and *mybody* are 2.5 to 3.5 and 5.1 to 5.9 respectively.

### Out-of-Body

**Fig 5:**
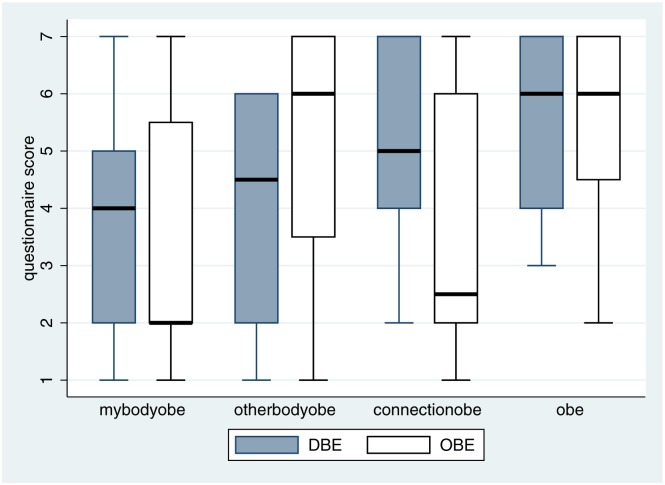
Box plots of the out-of-body questions by condition.

Fig 5 shows the box plots of questions asked after the out-of-body phase (Table 1). Overall participants in both groups affirmed that they had felt ‘out of their body’ (the variable ‘obe’). However, in line with previous results [31, 32] those in the DBE condition tended to affirm body ownership and connection with the virtual body that they saw below, whereas those in the OBE condition tended to disown the virtual body (*mybodyobe*, *otherbodyobe*, *connectionobe*). The results for the remaining questions are given in Section C in S1 File, where it can be seen that the scores for *floatingobe*, *elevatedobe*, and *invisibleobe* are very high, and not different between the two groups.

Statistical analysis is given in Table 3. The variable *connectionobe* is negatively associated with the OBE condition, and *otherbodyobe* is positively associated, with high posterior probabilities. The variables *connectionobe* and *mybodyobe* are highly correlated (Spearman’s rho = 0.67, n = 32) and *connectionobe* was used in the model since it gave the best overall fit.

**Table 3:** Results of the Statistical Analysis on all the responses with condition as the independent variable.

Response Variable, individual *i*	Linear Model	H	P(H|D)D = dataPriors = 0.0013	Interpretation
**connectionobe** *y*_*i*1_	*β*_10_ + *β*_11_X_*i*_	*β*_11_ < 0	0.98	Very strong evidence that *connectionobe* is less in OBE than DBE condition.
**otherbodyobe** *y*_*i*2_	*β*_20_ + *β*_21_X_*i*_	*β*_21_ > 0	0.98	Very strong evidence that *otherbodyobe* is greater in OBE than DBE condition.
**drop2** *y*_*i*3_	*drop1*_*i*_ + *β*_30_ + *β*_31_X_*i*_	*β*_31_ > 0	0.996	Overwhelming evidence that *drop2* is greater in OBE than DBE condition.
**solitude** *y*_*i*4_	*β*_40_ + *β*_41_X_*i*_	*β*_41_ < 0	0.94	Strong evidence that *solitude* is less in OBE than DBE condition.
**lifeisbrief** *y*_*i*5_	*β*_50_ + *β*_51_X_*i*_	*β*_51_ < 0	0.73	Some evidence that *lifeisbrief* is less in OBE than DBE condition.
**loseall** *y*_*i*6_	*β*_60_ + *β*_61_X_*i*_	*β*_61_ < 0	0.91	Strong evidence that *loseall* is less in OBE than DBE condition.
**dieyoung** *y*_*i*7_	*β*_70_ + *β*_71_X_*i*_	*β*_71_ < 0	0.80	Good evidence that *dieyoung* is less in OBE than DBE condition.
**howitwillbe** *y*_*i*8_	*β*_80_ + *β*_81_X_*i*_	*β*_81_ < 0	0.89	Good evidence that *howitwillbe* is less in OBE than DBE condition.
**candonothing** *y*_*i*9_	*β*_90_ + *β*_91_X_*i*_	*β*_91_ < 0	0.97	Strong evidence that *candonothing* is less in OBE than DBE condition.
**disintegration** *y*_*i*,10_	*β*_10,0_ + *β*_10,1_X_*i*_	*β*_10,1_ < 0	0.68	Little evidence that *disintegration* is less in OBE than DBE condition.

X is the experimental condition (X = 0 DBE, X = 1 OBE). H is the hypothesis on the parameter of interest. The distributions are shown in Section D in S1 File with further statistics in Section E in S1 File

### Mental Ball-Dropping Task

**Fig 6:**
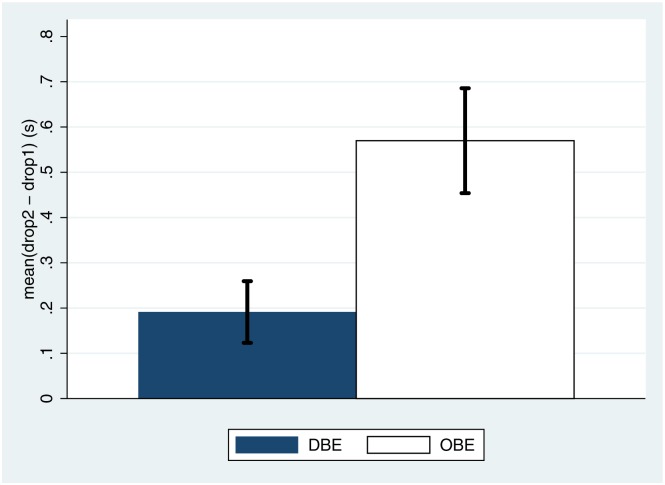
Mean and Standard Error of mean drop time during the out-of-body phase minus the mean drop time during the in-the-body phase, by condition.

The times between an auditory signal indicating to participants that they should drop an imaginary apple and it reaching the floor were measured five times at the end of each of the in-the-body and out-of-body phases, and the means recorded (*drop1*, *drop2*), as described earlier. Fig 6 shows the means and standard errors of *drop2*-*drop1*. The results reproduce earlier work [32] showing that the sense of location is shifted above and out of the body in the OBE compared to the DBE condition. The result in Table 3 shows that this has overwhelming posterior probability support.

### Fear of Death

**Fig 7:**
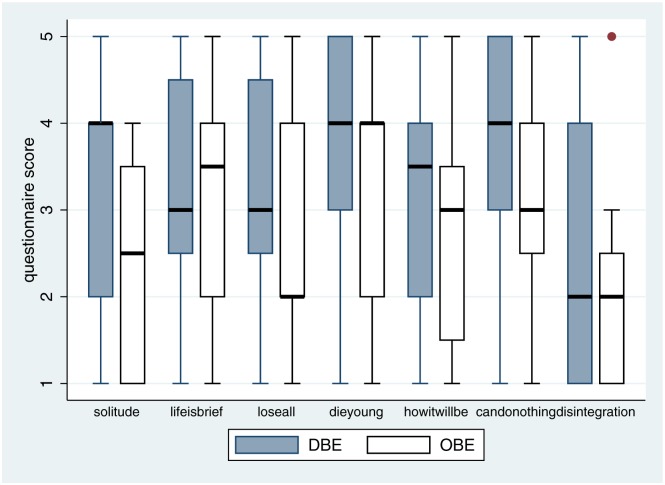
Box plots for the Fear of Death questionnaire responses.

First we consider the answers to the individual questions shown in Table 2. Box plots for the responses are shown in Fig 7. Taking into account both the medians and interquartile ranges it appears that responses tend to be lower in the OBE compared to the DBE condition, indicating less fear of death being associated with OBE. Table 3 shows that all of the posterior probabilities of OBE responses being less than DBE are greater than 0.5. The lowest probability is for *disintegration* and the greatest is for *candonothing*, as can be seen in Fig 7.

**Fig 8:**
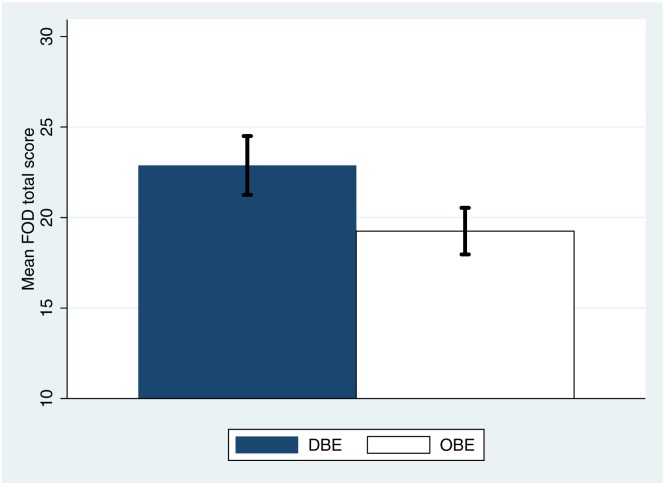
Bar Chart showing the means and standard errors of the total FOD score by condition. The minimum possible score is 7 and the maximum possible is 35.

**Fig 9:**
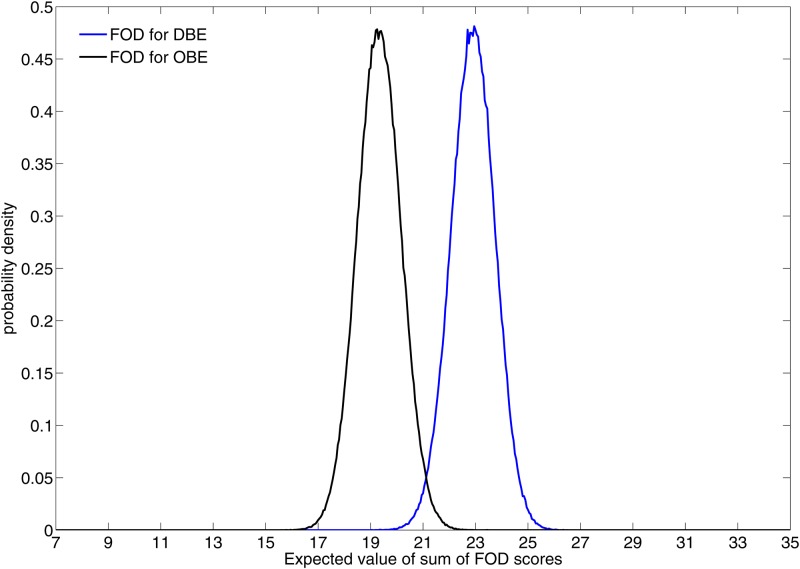
Posterior probability distributions of the expected values of the total FOD scores by Condition. The 95% credible intervals are 21.3 to 24.5 for DBE, and 17.7 to 21.0 for OBE.

Moving to the total score, Fig 8 shows that this is less in the OBE than the DBE condition. Fig 9 shows the posterior probability distributions of the expected values of the FOD total scores under the two conditions. This clearly shows a strong separation between the two conditions. For example, the posterior probability of a total FOD score of less than or equal to 21 is 0.012 in the DEB condition and 0.98 in the OBE condition.

**Fig 10:**
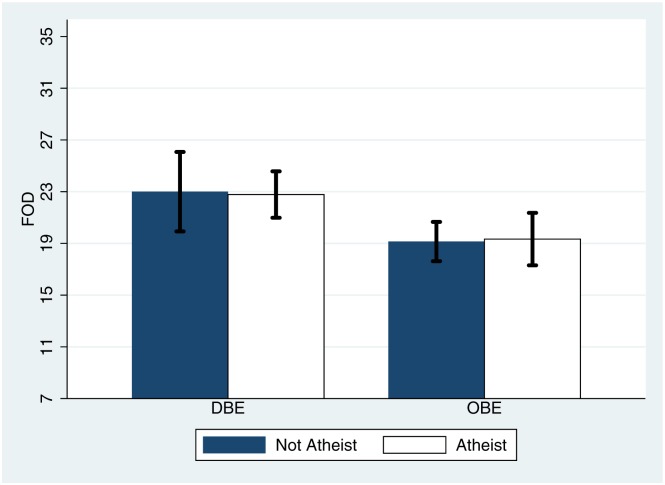
Bar Chart showing the means and standard errors of the total FOD score by condition and atheism. The minimum possible score is 7 and the maximum possible is 35.

It is possible that religious convictions of the participants, obtained from participants after the VR exposure, could play a role in FOD. Fig 10 shows the means and standard errors of FOD by condition and whether or not participants declared as atheists (9 in each condition). It is clear that there was no influence of religious conviction, at least at this basic level, on the results for FOD. However, a larger sample size and a more sensitive measure of religious or spiritual beliefs would be needed to explore this further.

## Discussion

There have been several articles showing that embodiment of participants in bodies with characteristics that are different from their own bodies can have perceptual and cognitive effects—for example, putting light-skinned people in dark-skinned bodies seems to reduce implicit racial bias—for a review see [24]. Also it has been found that if there is body ownership over a virtual body and then participants are lifted out of that body, there is a strong physiological response (Heart Rate Deceleration) to an attack seen on that body, with the HRD correlating with the questionnaire scores on body ownership [18]. In a recent study Pamment and Aspell [40] compared the two paradigms that we have adapted here to examine their influence on chronic pain. They found that patients’ ratings of pain substantially reduced during a virtual out-of-body experience, but that there was no overall difference in ratings between the two OBE paradigms. Moreover using the method of inducing an out-of-the-body experience on which our OBE condition was based [27], it was shown by Bergouignan, Nyberg, et al. [41] that episodic memory is impaired (becoming spatially and temporally fragmented) in the OBE condition compared with memory of the same event experienced from the normal body perspective. Here we have shown that there may also be further psychological effects due to a virtual out-of-body experience with respect to a previously owned virtual body, in particular in relation to death anxiety.

First, we showed that using a combination of first person perspective, visuomotor and visuotactile synchronous stimulation we were able to induce a high level of subjective body ownership during an embodied phase (in-the-body). Next we showed that our experimental condition that induced an out-of-body experience where the participant’s visual and visuotactile perception was shifted up and out of the virtual body (OBE) resulted in disownership of the previously owned body in line with previous research [31]. In our control condition (DBE) perception is divided between visuotactile and visuomotor integration on the body seen below, but with visual centre of location up and out of the body. This resulted in a maintained feeling of connection with the virtual body. That there was a shift in self-location up and out of the body in the OBE but less in the DBE was further demonstrated by the results of the ‘mental ball-dropping task’. All these results are in line with [32]. What is new is that we showed that in the OBE condition the overall fear of death is lower than in the DBE condition.

Out-of-body experiences are usually reported in the context of near-death experiences, but they also occur spontaneously. There is not a lot of previous work on the life-consequences of OBEs that occur independently of NDEs. However, in a survey of 339 respondents to a questionnaire about out-of-body experiences Twemlow, Gabbard, et al. [42] reported that some time after their experience 84% wanted to have the experience again, 81% felt that the experience was very pleasant, and 71% said that it was of lasting benefit. Most importantly for our discussion, 63% reported that it enhanced their belief towards life after death, compared to 32% of a control group (n = 81 people who were interested in OBEs but had never experienced one). See also similar results reported in [43, 44].

Our results are consistent with such empirical findings as there are, that people who have had spontaneous OBEs, not necessarily in the context of a NDE, are also likely to have reduced death anxiety. Metzinger [10] argued that an OBE is like a “globalized phantom-limb experience” where there are two bodies—the physical body seen from the outside and a more ethereal body (the phantom). In an OBE this ethereal body is perceived as being spatially located outside of the physical body. If this ethereal body is conflated with the idea of ‘soul’ then this provides folk-phenomenological evidence that indeed the self can exist outside of the physical body, that this constitutes the soul—and that therefore, we would add, there is the possibility of survival beyond physical death, since in this view conscious existence may not depend on the physical body. The virtual reality experience provides an experiential but *implicit* learning of this possibility for consciousness to exist outside of the body, but without the implication that participants would explicitly believe this. Here by consciousness we refer to the centre of perceptual consciousness—that is the location at which we are aware of perceiving multisensory information that locates us in space. In our OBE condition visual and visuotactile integration were located out of the (previously owned) virtual body. In the DBE condition there is still substantial sensory data that appears to be centered on the virtual body. In this case consciousness in the sense of the multisensory information that indicates self-location is split between two different positions. This experience therefore does not provide clear evidence of the possibility for perceptual consciousness to exist outside the body.

In our experiment participants did not know prior to their experience that the study was in any way related to death anxiety. Our results open up the possibility that the virtual OBE experience provides an implicit learning that consciousness in the sense of the centre of perception can be separate from the physical body, and that therefore death of the physical body is not necessarily the end of consciousness. In further work we put this in the context of near-death experiences in virtual reality, with longer term follow-up of the effects, and examine the possibility of broader life-changes than those only related to fear of death.

## Methods

Participants were recruited between December 2014 and March 2015, using the webpage of the Department of Basic Psychology (http://www.ub.edu/psicologiabasica) of the University. They received two course credit points for participating. Participants had to be female, aged at least 18, not suffering from epilepsy, and not planning to drive a vehicle for 3 hours after the termination of the experiment. The number of participants who completed the experiment and for which data were collected was 32. Twelve others who had been recruited did not complete the experiment, 1 because of dizziness, 10 because of equipment or software failure during the experiment, and 1 could not understand the mental ball-dropping task.

The Virtual Environment (VE) was designed and rendered using Unity3D (Unity Technologies, Copenhagen, Denmark) and consisted of a room with some standard furniture. It was displayed in an Oculus head-mounted-display (HMD; Oculus VR, Irvine, California, United States) at 75Hz using a 1080p OLED panel, split to 75 Hz 960×1080 per eye rendering. Head tracking was achieved using the HMD’s internal head tracking. Additionally an Intersense Wand joystick connected to an Intersense IS-9006 hybrid inertial/acoustic 6-DOF tracking system (Intersense, Billerica, Massachusetts, United States) with data streamed over a VRPN network [45] to the PC running the application. Button clicks on this were used for measuring the estimated drop times in the MBD task [32]. The virtual body used was a 3D model of a female character from Rocketbox Studios (Hannover, Germany). Participant movements (hands and feet) were tracked by a Natural Point’s Optitrack infrared system (NaturalPoint, Inc. Corvallis, United States), consisting of 12 cameras, tracking 3 triplets of reflective markers on each foot, wrist and forearm of the participant. Positions and orientations of the markers were calculated in real-time and streamed to the virtual reality application by Natural Point’s Tracking Tools software (NaturalPoint, Inc. Corvallis, United States) using the VRPN protocol [45]. An inverse kinematic algorithm was applied to map the movement onto the virtual body [46].

Tactile stimulation was administered by means of 4 vibrotactile devices fastened on both wrists and ankles of the participant and controlled by an Arduino (Arduino srl., Ivrea, Italy) board connected to the computer with a USB cable [46]. The vibrotactile devices vibrated for 100ms at a frequency of 150Hz. Tactile feedback was activated on the area of the participant’s body corresponding to where the virtual balls hit the virtual body.

The study was divided into two phases, an initial embodiment phase (in-the-body) that was the same in both groups and the out-of-body phase which was different for each group. In total the experiment lasted between 30–40 min, including 15 minutes in the VR.

After being instructed about the experimental procedures and after they signed the consent form, participants were asked to put on a black tracking suit and black socks. Their limbs were measured in order to scale their virtual body so that it better fitted with their real body proportions. Participants were seated in a chair with their arms on their thighs and their legs lying stretched on a coffee table (Fig 1A). Then vibrators were attached to participant’s wrists and ankles, and tracking markers were collocated on their limbs. They were instructed about the experimental procedures and before putting the HMD, use of the Wand trigger was explained. During the VR experience a recorded voice gave instructions.

When looking down at themselves participants saw a virtual body from a first person perspective (1PP) in the same posture and position as their real body, wearing black clothes (Fig 1B). They could see this body by directly looking towards themselves and in a virtual mirror to their front. In the virtual mirror they would see that their virtual body was also wearing an Oculus. Due to the real-time motion capture the virtual body moved congruently with their real movements (S1 Video).

Each experimental session started with the participant being asked to describe the VE in order to explore and become familiar with it. This was followed by the training for the Mental Ball-dropping task (MBD). They were asked to estimate the time an imaginary apple would take to fall from their left hand to the floor. Participants had to click a button of the Wand twice, first when releasing the imaginary apple and second when they estimated the apple would reach the floor [32]. The times of both button presses were recorded. They were trained on the MBD task at the beginning of the experiment (just after entering VR) for three trials, where they learned that an auditory cue signaled a trial of the MBD task.

After the MBD training phase the voice asked participants to look towards their legs and the table on which they rested and follow with their feet the path that appeared on the table, following the method described in [19]. This was to induce body ownership through synchronous visuomotor stimulation. This was followed by synchronous visuotactile stimulation where virtual balls struck the virtual body while participants felt corresponding vibrotactile stimulation. It is important to note that visuomotor and visuotactile stimulation were combined. The balls would strike the same places on the hands and feet even if these were moving. Next the mean MBD baseline was recorded (*drop1*) from 5 repetitions of this task. After this first embodiment phase participants were asked two questions about body ownership (Table 1).

The HMD faded to black between the two phases. In the second phase at first there was another period of synchronous visuomotor and visuotactile stimulation with the balls striking the virtual body. Then the viewpoint of the participant was lifted out of the virtual body towards the ceiling of the virtual room, and just behind the body, so that the body could be seen below. In the DBE condition, while balls kept striking the virtual body that participants saw below, they continued feeling the vibrations on their real body. In addition, real body movements caused corresponding movements on the virtual body below. In the OBE condition, the balls continued to strike the area corresponding to where the body would be in relation to the elevated viewpoint rather than the virtual body below. Real body movements were not translated into movements on the virtual body below. After this, in the same position, for both conditions, participants completed the MBD task five times and the durations were recorded (with mean *drop2*). Then the displays faded to black and participants were asked the same two questions as before together with questions related to the OBE experience (Table 2). Then after removing trackers and vibrators with the minimum interaction possible, participant were asked to complete the Fear of Death questionnaire and were debriefed about the purpose of the experiment.

## Supporting Information

S1 File Supporting Information. (A) Participant information, (B) Statistical Model (C) Further out-of-body questions, (D) Posterior distributions of the model parameters (E) Statistics of the posterior distributions of the model parameters.
(DOCX)

S1 Data An Excel Spreadsheet. This excel spreadsheet contains the original data. The comments in the header column explain the meaning of each variable.
(XLSX)

S1 Video Supporting Video. A video illustrating the experimental procedures and conditions.
(MP4)

## References

[1] Parnia S, Waller DG, Yeates R, Fenwick P. A qualitative and quantitative study of the incidence, features and aetiology of near death experiences in cardiac arrest survivors. Resuscitation. 2001;48(2):149–56. 11426476 10.1016/s0300-9572(00)00328-2

[2] Blanke O, Dieguez S. Leaving body and life behind: Out-of-body and near-death experience In: Laureys S, T G, editors. The neurology of consciousness: Cognitive neuroscience and neuropathology: Elsevier Academic Press; 2009 p. 303–25.

[3] Agrillo C. Near-death experience: out-of-body and out-of-brain? Rev Gen Psychol. 2011;15(1):1–10.

[4] Moody RA. Life After Life: The investigation of a phenomenon, survival of bodily death: Random House; 2001.

[5] Blackmore SJ. Beyond the body: An investigation of out-of-the-body experiences: Academy Chicago Publishers; 1982.

[6] Van Lommel P, van Wees R, Meyers V, Elfferich I. Near-death experience in survivors of cardiac arrest: a prospective study in the Netherlands. The Lancet. 2001;358:2039–45. 10.1016/S0140-6736(01)07100-8 11755611

[7] Klemenc-Ketis Z. Life changes in patients after out-of-hospital cardiac arrest. Int J Behav Med. 2013;20(1):7–12. 10.1007/s12529-011-9209-y 22161220

[8] Parnia S, Spearpoint K, Fenwick PB. Near death experiences, cognitive function and psychological outcomes of surviving cardiac arrest. Resuscitation. 2007;74(2):215–21. 10.1016/j.resuscitation.2007.01.020 17416449

[9] Blanke O, Landis T, Spinelli L, Seeck M. Out-of-body experience and autoscopy of neurological origin. Brain. 2004;127:243–58. 10.1093/brain/awh040 14662516

[10] Metzinger T. Out-of-body experiences as the origin of the concept of a 'soul'. Mind and Matter. 2005;3(1):57–84.

[11] Kilteni K, Groten R, Slater M. The Sense of Embodiment in Virtual Reality. Presence: Teleoperators and Virtual Environments. 2012;21:373–87.

[12] Botvinick M, Cohen J. Rubber hands 'feel' touch that eyes see. Nature. 1998;391(6669):756-. 9486643 10.1038/35784

[13] Armel KC, Ramachandran VS. Projecting sensations to external objects: evidence from skin conductance response. Proc R Soc Lond B. 2003;270:1499–506. 10.1098/rspb.2003.2364 PMC1691405 12965016

[14] Ehrsson HH, Wiech K, Weiskopf N, Dolan RJ, Passingham RE. Threatening a rubber hand that you feel is yours elicits a cortical anxiety response. PNAS. 2007;104(23):9828–33. 10.1073/pnas.0610011104 17517605 PMC1887585

[15] Petkova VI, Ehrsson HH. If I Were You: Perceptual Illusion of Body Swapping. PLoS One. 2008;3:e3832 10.1371/journal.pone.0003832 19050755 PMC2585011

[16] Slater M, Perez-Marcos D, Ehrsson HH, Sanchez-Vives MV. Towards a digital body: The virtual arm illusion. Front Hum Neurosci. 2008;2. 10.3389/neuro.09.006.2008 PMC2572198 18958207

[17] González-Franco M, Peck TC, Rodríguez-Fornells A, Slater M. A threat to a virtual hand elicits motor cortex activation. Exp Brain Res. 2013;232(3):875–87. 10.1007/s00221-013-3800-1 24337257

[18] Slater M, Spanlang B, Sanchez-Vives MV, Blanke O. First person experience of body transfer in virtual reality. PLoS One. 2010;5(5):e10564–e. 10.1371/journal.pone.0010564 20485681 PMC2868878

[19] Kokkinara E, Slater M. Measuring the effects through time of the influence of visuomotor and visuotactile synchronous stimulation on a virtual body ownership illusion. Perception. 2014;43(1):43–58. 24689131 10.1068/p7545

[20] Maselli A, Slater M. The building blocks of the full body ownership illusion. Front Hum Neurosci. 2013;7. 10.3389/fnhum.2013.00083 PMC3604638 23519597

[21] Maselli A, Slater M. Sliding Perspectives: dissociating ownership from self-location during full body illusions in virtual reality. Front Hum Neurosci. 2014;8:693 10.3389/fnhum.2014.00693 25309383 PMC4161166

[22] Peck TC, Seinfeld S, Aglioti SM, Slater M. Putting yourself in the skin of a black avatar reduces implicit racial bias. Conscious Cogn. 2013;22:779–87. 10.1016/j.concog.2013.04.016 23727712

[23] Banakou D, Groten R, Slater M. Illusory ownership of a virtual child body causes overestimation of object sizes and implicit attitude changes. PNAS. 2013;110:12846–51. 10.1073/pnas.1306779110 23858436 PMC3732927

[24] Maister L, Slater M, Sanchez-Vives MV, Tsakiris M. Changing bodies changes minds: owning another body affects social cognition. Trends Cogn Sci. 2015;19(1):6–12. 10.1016/j.tics.2014.11.001 25524273

[25] Kilteni K, Maselli A, Koerding K, Slater M. Over my fake body: body ownership illusions for studying the multisensory basis of own-body perception. Name: Frontiers in Human Neuroscience. 2015;9:141 10.3389/fnhum.2015.00141 25852524 PMC4371812

[26] Blanke O, Slater M, Serino A. Behavioral, Neural, and Computational Principles of Bodily Self-Consciousness. Neuron. 2015;88(1):145–66. 10.1016/j.neuron.2015.09.029 26447578

[27] Ehrsson HH. The Experimental Induction of Out-of-Body Experiences. Science. 2007;317:1048 10.1126/science.1142175 17717177

[28] Hiromitsu K, Midorikawa A. Downward and Parallel Perspectives in an Experimental Study of Out-of-Body Experiences. Multsensory Research. 2016;29(4–5):439–51. 10.1163/22134808-00002523 29384611

[29] Lenggenhager B, Tadi T, Metzinger T, Blanke O. Video ergo sum: Manipulating bodily self-consciousness. Science. 2007;317:1096–9. 10.1126/science.1143439 17717189

[30] Pomes A, Slater M. Drift and ownership toward a distant virtual body. Front Hum Neurosci. 2013;7. 24399960 10.3389/fnhum.2013.00908 PMC3872309

[31] Guterstam A, Ehrsson HH. Disowning one's seen real body during an out-of-body illusion. Conscious Cogn. 2012;21:1037–42. 10.1016/j.concog.2012.01.018 22377139

[32] Lenggenhager B, Mouthon M, Blanke O. Spatial aspects of bodily self-consciousness. Conscious Cogn. 2009;18:110–7. 10.1016/j.concog.2008.11.003 19109039

[33] Guterstam A, Abdulkarim Z, Ehrsson HH. Illusory ownership of an invisible body reduces autonomic and subjective social anxiety responses. Sci Rep. 2015;5(9831). 10.1038/srep09831 PMC4407500 25906330

[34] Lester D. The Collett-Lester fear of death scale: Taylor & Francis; 1994 45–60 p.

[35] Lester D, Abdel-Khalek A. The Collett-Lester fear of death scale: A correction. Death Stud. 2003;27(1):81–5. 10.1080/07481180302873 12508829

[36] Tomás-Sábado J, Limonero JT, Abdel-Khalek AM. Spanish adaptation of the Collett-Lester fear of death scale. Death Stud. 2007;31(3):249–60. 10.1080/07481180601152625 17330362

[37] Bergström I, Kilteni K, Slater M. First-person Perspective Virtual Body Posture Influences Stress: A virtual reality body ownership study. PLoS One. 2016;11(2): e0148060 10.1371/journal.pone.0148060 26828365 PMC4734707

[38] Plummer M, editor JAGS: A program for analysis of Bayesian graphical models using Gibbs sampling. Proceedings of the 3rd international workshop on distributed statistical computing; 2003; Vienna, Austria: Technische Universit at Wien.

[39] Banakou D, Slater M. Body Ownership Causes Illusory Self-Attribution of Speaking and Influences Subsequent Real Speaking. PNAS. 2014;111(49):17678–83. 10.1073/pnas.1414936111 25422444 PMC4267370

[40] Pamment J, Aspell J. Putting pain out of mind with an ‘out of body’illusion. European Journal of Pain. 2016. 10.1002/ejp.927 27509229

[41] Bergouignan L, Nyberg L, Ehrsson HH. Out-of-body–induced hippocampal amnesia. Proceedings of the National Academy of Sciences. 2014;111(12):4421–6. 10.1073/pnas.1318801111 PMC3970513 24616529

[42] Twemlow SW, Gabbard GO, Jones FC. The out-of-body experience: A phenomenological typology based on questionnaire responses. Am J Psychiatry. 1982;139(4):450–5. 10.1176/ajp.139.4.450 7039367

[43] Gabbard GO, Twemlow SW. With the eyes of the mind: An empirical analysis of out-of-body states: Praeger Publishers; 1984.

[44] Alvarado CS. Out-of-body experiences In: Cardeña E, Lynn SJ, Krippner S, editors. Varieties of anomalous experience: Examining the scientific evidence. Washington, D.C.: American Psychological Association; 2000.

[45] Taylor II RM, Hudson TC, Seeger A, Weber H, Juliano J, Helser AT. VRPN: a device-independent, network-transparent VR peripheral system. VRST '01: Proceedings of the ACM symposium on Virtual reality software and technology; New York, NY, USA: ACM Press; 2001. p. 55–61.

[46] Spanlang B, Normand J-M, Giannopoulos E, Slater M. A First Person Avatar System with Haptic Feedback ACM Symposium on Virtual Reality Software and Technology (VRST 2010); Hong Kong: ACM; 2010. p. 47–50.

