# Supplementary material for: A Virtual Out-of-Body Experience Reduces Fear of Death
Source: PLoS One. 2017 Jan 9;12(1):e0169343. doi: 10.1371/journal.pone.0169343 (PMC5221792; doi:10.1371/journal.pone.0169343)
Supplement: S1 File — (A) Participant information, (B) Statistical Model (C) Further out-of-body questions, (D) Posterior distributions of the model parameters (E) Statistics of the posterior distributions of the model parameters. (DOCX) [file pone.0169343.s001.docx]

A Virtual Out-of-Body Experience Reduces Fear of Death

Pierre Bourdin, Itxaso Barberia, Ramon Oliva, Mel Slater

Supporting Information

# A. Participants

**Table A** – Characteristics of the Participants by Experimental Group

|  | DBE | OBE |
| --- | --- | --- |
| **Age**: Mean ± SE | 20.1 ± 0.50 | 20.6 ± 0.56 |
| **Self Esteem^a^**: Median (IQR) | 35 (5) | 35 (4) |
|  |  |  |
| **Religion** | **Frequency** | |
| Believer and practicing | 1 | 0 |
| Believer non-practicing | 4 | 4 |
| Agnostic | 1 | 3 |
| Atheist | 9 | 9 |
| Other | 1 | 0 |

^a^ Self Esteem using the Rosenberg Self Esteem Scale (Rosenberg, 1965) with Spanish translation (Martín-Albo et al., 2007). There are 10 items each scored on a scale of 1, 2, 3 or 4. Taking the sum of these the maximum score is 40. The higher the overall score the greater the self-esteem.

# B. Statistical Model

This section is very similar to the method used in a previous paper (Bergström et al., 2016). The (Bayesian) statistical model is one overall model, where all equations are treated simultaneously rather than as a series of separate models. In other words the Bayesian method returns the joint posterior distribution of all the model parameters. In the following
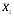
 refers to the Condition for the *i*th individual where $X_{i}=0$(DBE) or 1 (OBE). The overall model has the following components:

The **questionnaires scores**, *mybody* and *otherbody* do not depend on Condition (since they are recorded before the two conditions DBE and OBE are introduced). We use the logistic model in (Lunn et al., 2012) (p132-134). The probabilities
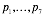
 of a score 1,…,7 respectively have prior distributions with vary wide variance. The expected values of Fig. 4 are computed from the distribution of the posterior expected values
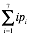
.

For the remaining questions in Tables 1-2 the parameters of the linear model that relate the mean of the logistic distribution to the linear model are specified as follows:
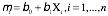
with prior distribution
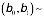
 bivariate normal with mean (0,±120) and variance-covariance matrix with each variance 1600 and each covariance 160. The mean for
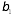
is taken as -120 in the case where our hypothesis is that
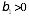
(e.g., otherbodyobe) and 120 when the hypothesis is that
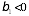
(e.g., connectionobe). Note that this gives the prior
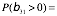
0.0013 (the probability of a standard normal variate being > 3) in the case when the mean is -120, and similarly
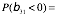
0.0013 when the mean is 120.

For the ***drop2* mean** the model is as shown in Table 3, where
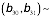
bivariate normal with variance-covariance matrix as above and mean for
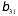
as -120 (since the hypothesis is that
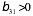
). The prior distribution of the variance of *drop2* was modeled as a Gamma distribution with parameters (0.001, 0.001) in the JAGS / BUGS specification.

For the total FOD (Fig. 9) the distribution of the sum of the expected values of each of the 7 components (shown in Table 3) was found. The individual expected value distributions were modeled as in 1 above.

Under this method readers are free to interpret the probabilities of the hypotheses in different ways of course. We have used the following: We start with a strong bias against each of the hypotheses - the prior probability assigned is about 1/1000. If the posterior probabilities are around the 50% range then we would say that from being biased against the hypothesis we move to a 50-50 probability and more evidence is needed. Probabilities above 70% we refer to as ‘some’ evidence in favor of the hypothesis. For 80% or more we use the term ‘good evidence’. Above 90% ‘strong evidence’, and in one case with the probability almost 1 we use the terms ‘very strong’ or ‘overwhelming evidence’.

Each Markov Chain Monte Carlo simulation was run 7 times (according to convention) with a sample size of 60,000 observations and a burn-in of 3000. All Rhat values - measuring consistency between the results of the 7 chains - were equal to 1.0 (i.e., to 1 d.p.) meaning that reasonable convergence was obtained.

# C. Further out-of-body questions

Figure A shows the out-of-body questions not included in Fig. 5.


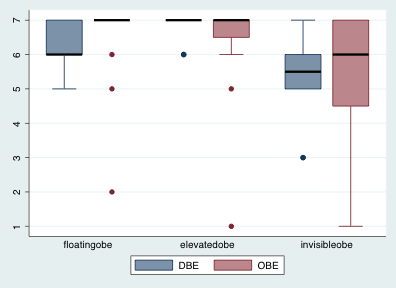


Fig. A - Box plots for the out-of-body questions additional to those of Fig. 5 (see Table 1)

# D. Posterior distributions of the model parameters

The following Figures should be examined in relation to Table 3 and Section E below, they give the posterior distributions of the model parameters.


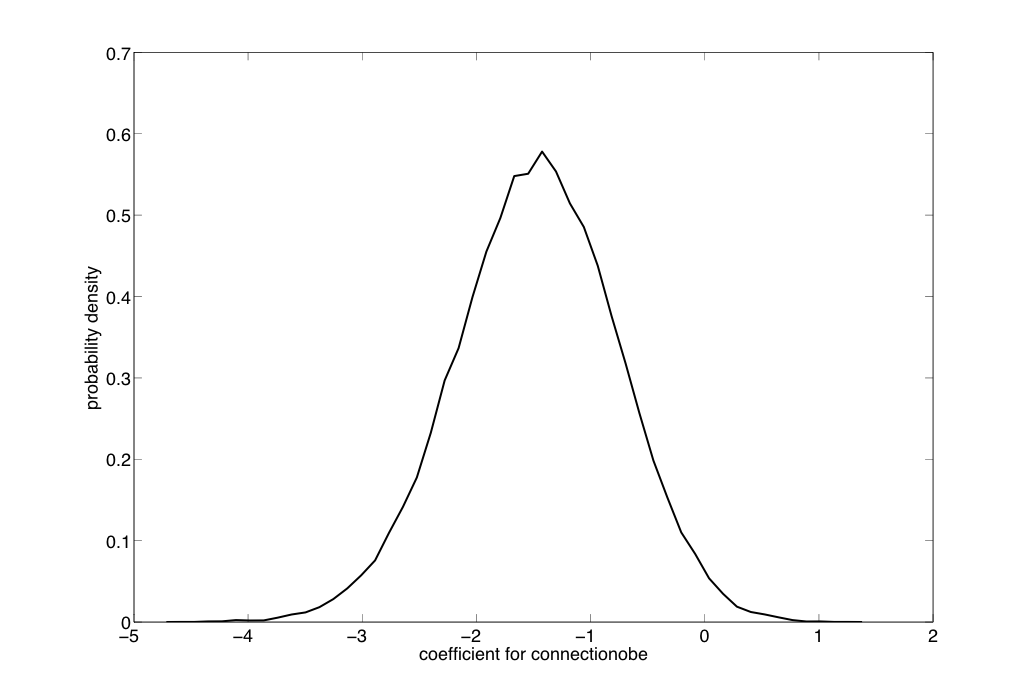


Figure B - Posterior distribution of the coefficient of Condition
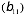
 in the model for *connectionobe*.


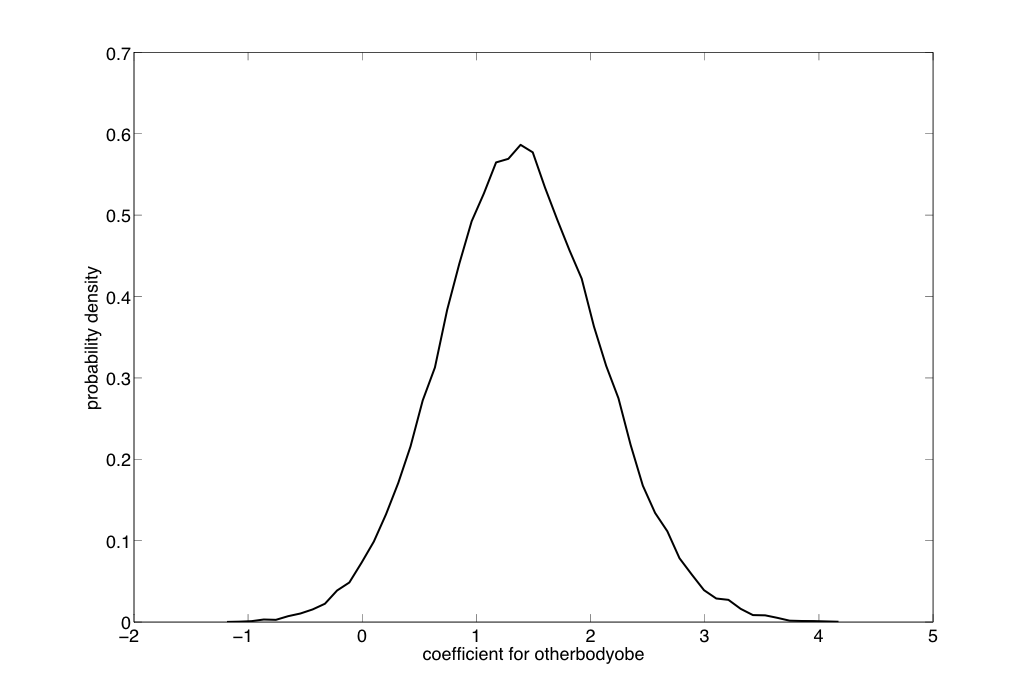
Figure C - Posterior distribution of the coefficient of Condition
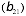
 in the model for *otherbodyobe*.


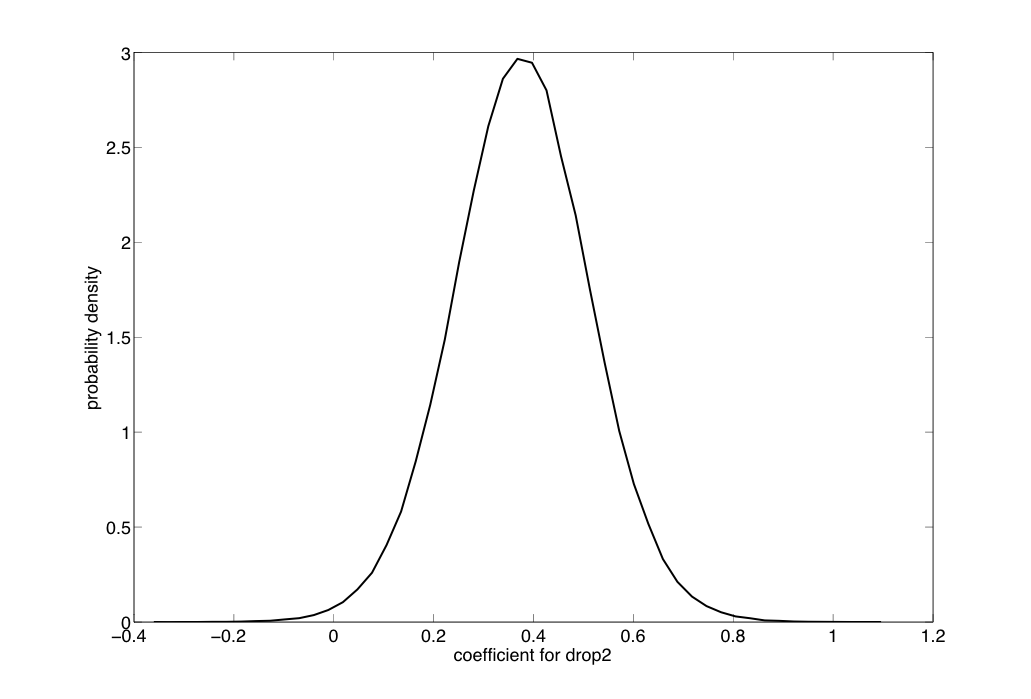


Figure D - Posterior distribution of the coefficient of Condition
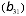
 in the model for *drop2*.


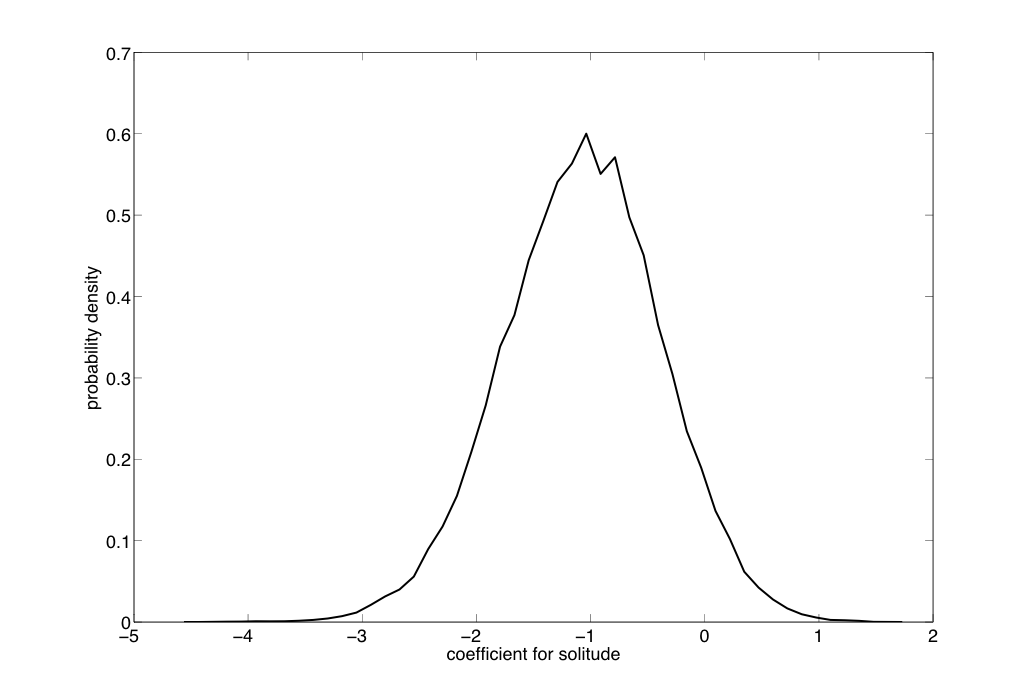


Figure E - Posterior distribution of the coefficient of Condition
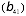
 in the model for *solitude*.


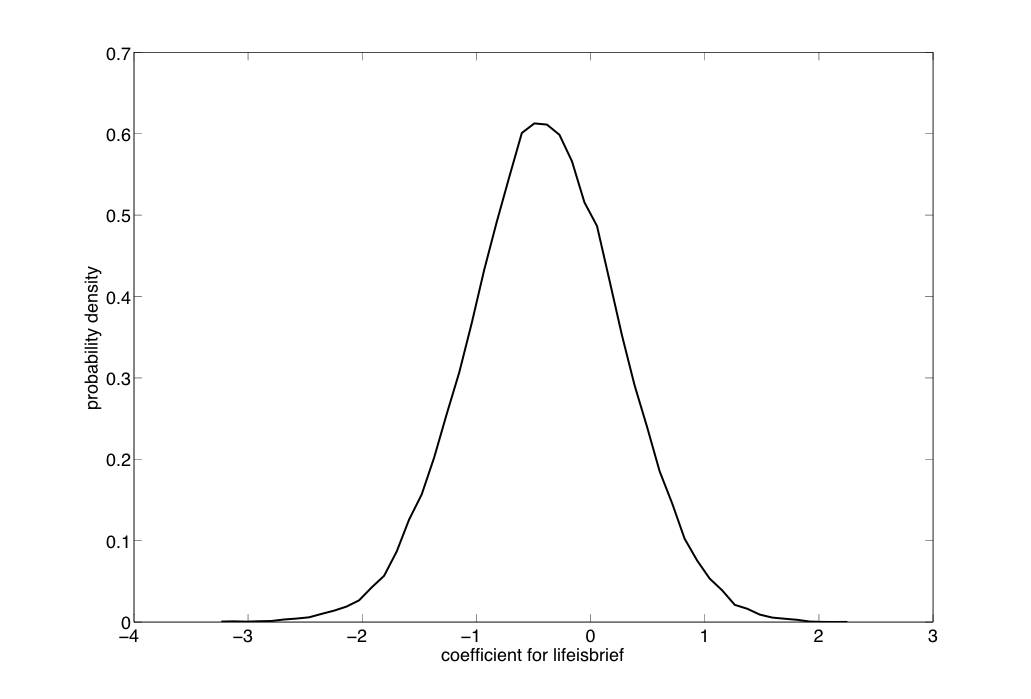


Figure F - Posterior distribution of the coefficient of Condition
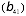
 in the model for *lifeisbrief*.
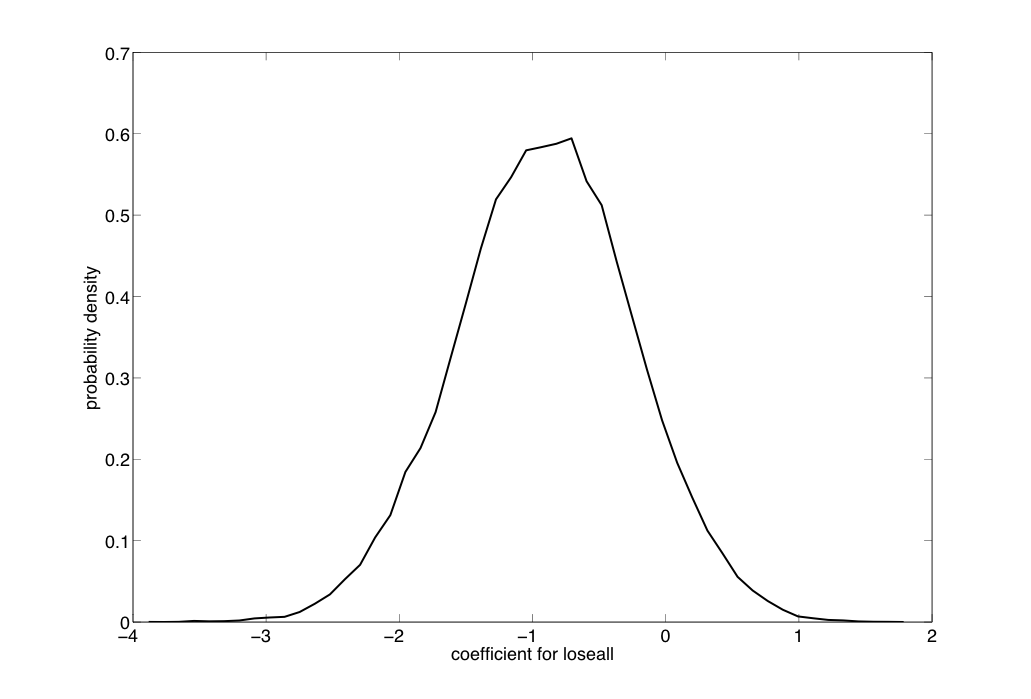


Figure G - Posterior distribution of the coefficient of Condition
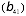
 in the model for *loseall*.


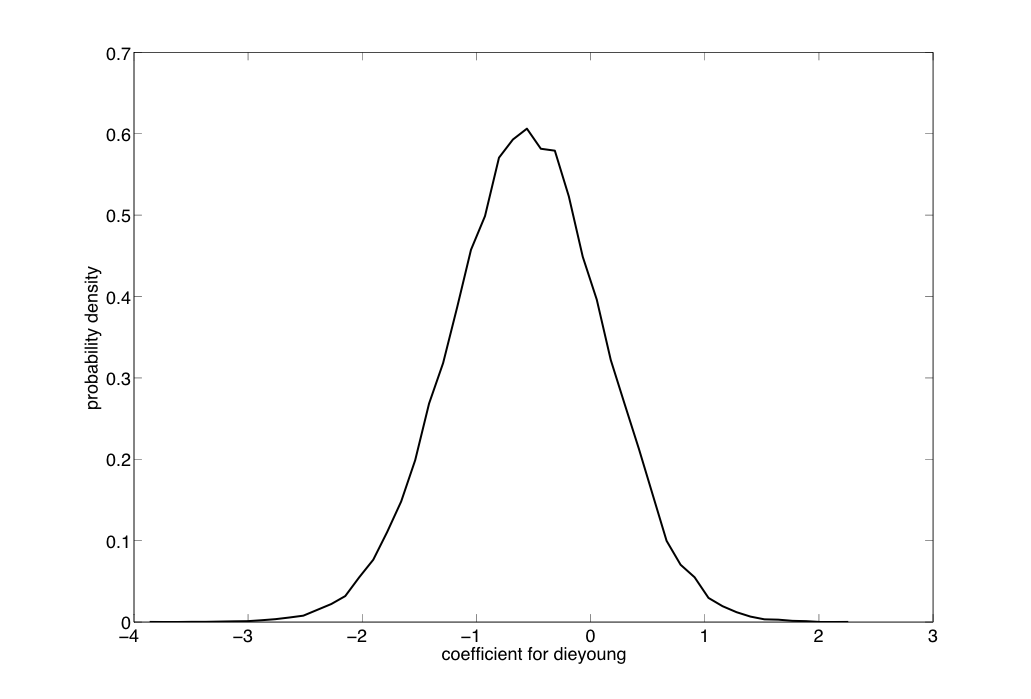


Figure H - Posterior distribution of the coefficient of Condition
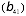
 in the model for *dieyoung*.


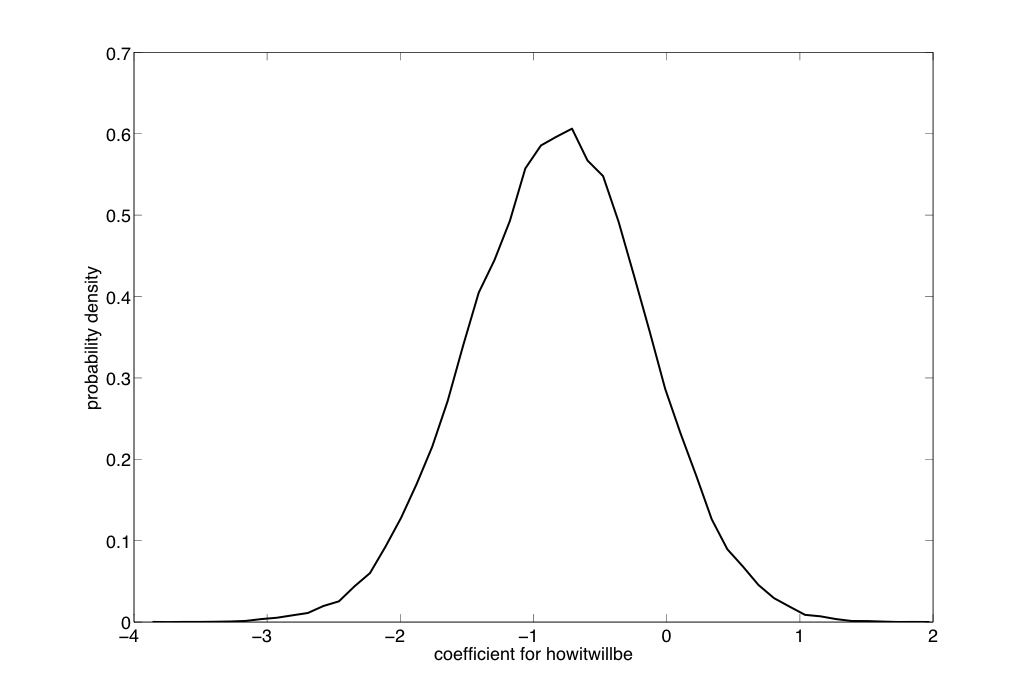


Figure I - Posterior distribution of the coefficient of Condition
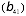
 in the model for *howitwillbe*.


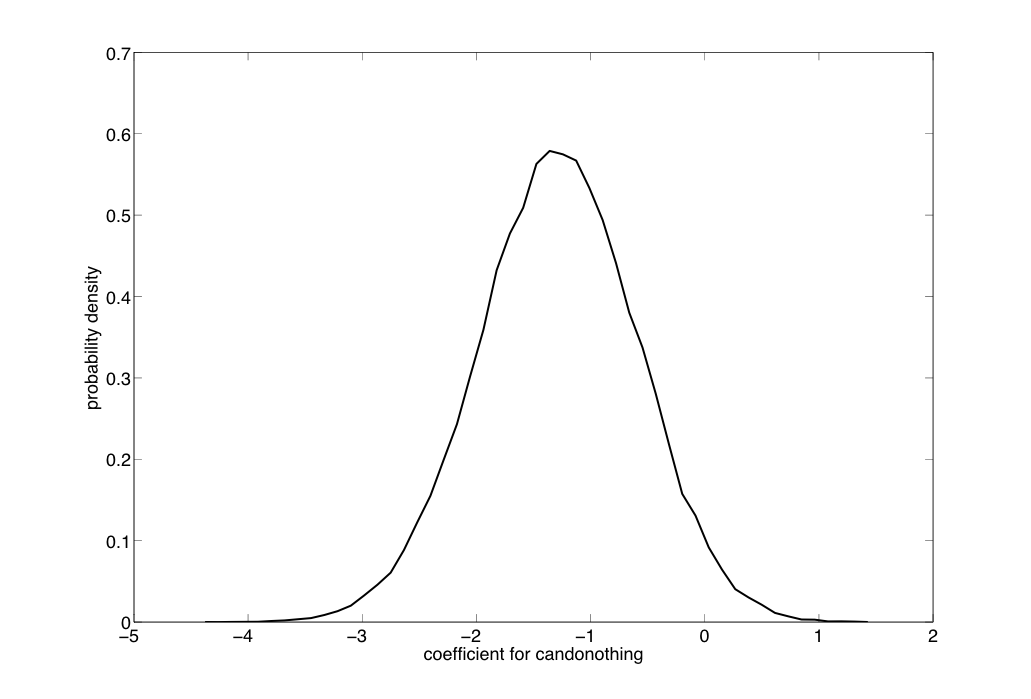


Figure J - Posterior distribution of the coefficient of Condition
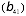
 in the model for *candonothing*.


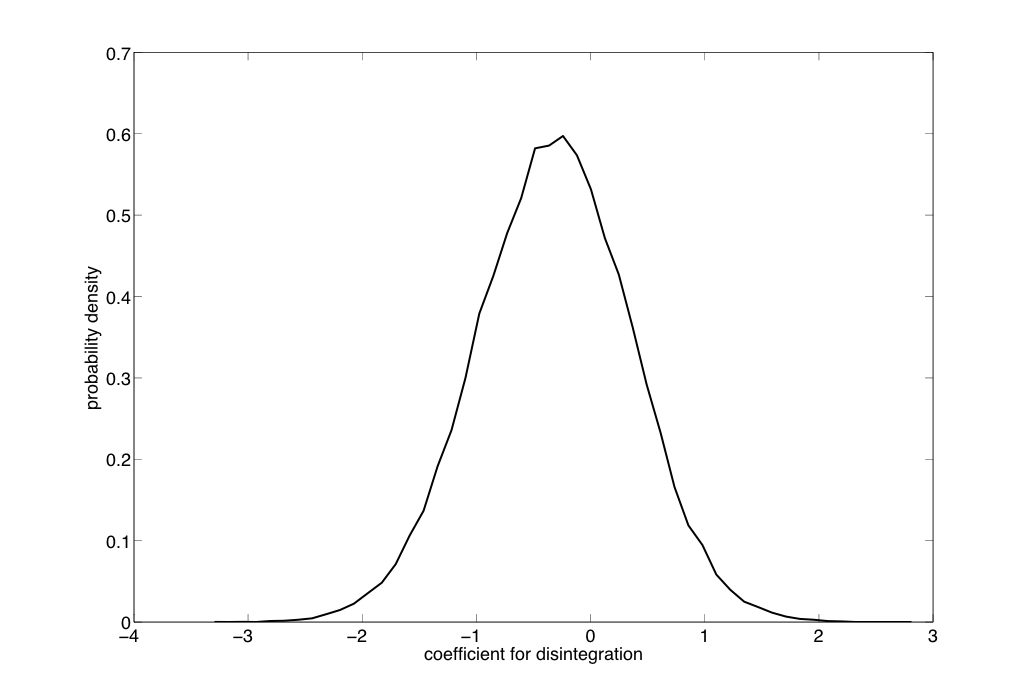


Figure K - Posterior distribution of the coefficient of Condition
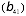
 in the model for *disintegration*.


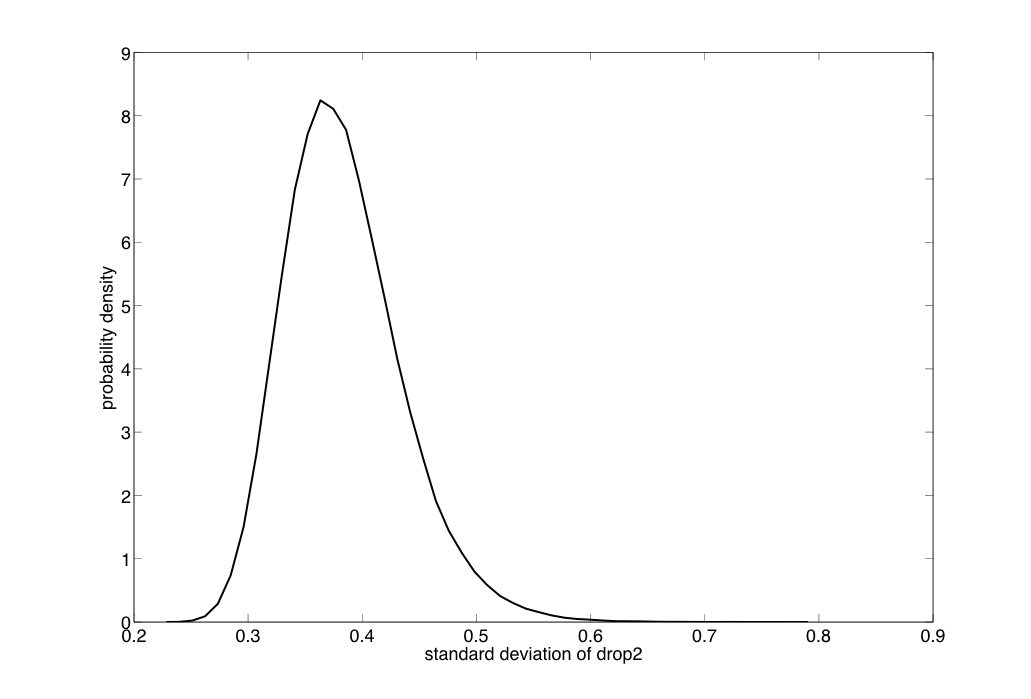


Figure L - Posterior distribution of the standard deviation of *drop2*.

# E. Statistics of the Posterior Distributions of the Parameters

Table B shows the mean, SD and 95% credible intervals of the posterior distributions of the parameters in Section D.

**Table B** - Mean, SD and 95% Credible Intervals for the Posterior Distribution of Coefficient of Condition in Table 3.

| **Coefficient of Condition** | **Mean** | **SD** | **95% Credible Interval** |
| --- | --- | --- | --- |
| connectionobe | -1.5 | 0.71 | -2.9 to -0.1 |
| otherbodyobe | 1.4 | 0.69 | 0.1 to 2.8 |
| drop2 | 0.4 | 0.13 | 0.1 to 0.6 |
| solitude | -1.1 | 0.69 | -2.5 to 0.2 |
| lifeisbrief | -0.4 | 0.66 | -1.7 to 0.9 |
| loseall | -0.9 | 0.67 | -2.2 to 0.4 |
| dieyoung | -0.6 | 0.66 | -1.9 to 0.7 |
| howitwillbe | -0.8 | 0.66 | -2.1 to 0.5 |
| candonothing | -1.3 | 0.69 | -2.7 to 0.0 |
| disintegration | -0.3 | 0.68 | -1.7 to 1.0 |

# Supporting References

Bergström, I., Kilteni, K., and Slater, M. (2016). First-person Perspective Virtual Body Posture Influences Stress: A virtual reality body ownership study. *PLOS ONE* 11(2): e0148060.

Lunn, D., Jackson, C., Best, N., Thomas, A., and Spiegelhalter, D. (2012). *The BUGS book: A practical introduction to Bayesian analysis.* CRC press.

Martín-Albo, J., Núñez, J.L., Navarro, J.G., and Grijalvo, F. (2007). The Rosenberg Self-Esteem Scale: translation and validation in university students. *The Spanish journal of psychology* 10**,** 458-467.

Rosenberg, M. (1965). *Society and the adolescent self-image.* Princeton, N.J., USA: Princeton University Press.
